# Shared autonomous HERV loci transcription identifies a unique circulating CD14+-xCR1+ mononuclear cell phenotype in a patient group with post-acute sequelae of COVID-19

**DOI:** 10.1371/journal.pone.0349350

**Published:** 2026-05-19

**Authors:** Hyunmin Koo, Casey D. Morrow

**Affiliations:** 1 Department of Genetics, Hugh Kaul Precision Medicine Institute, Heersink School of Medicine Immunology Institute, University of Alabama at Birmingham, Birmingham, Alabama, United States of America; 2 Department of Cell, Developmental and Integrative Biology, Hugh Kaul Precision Medicine Institute, Heersink School of Medicine Immunology Institute, University of Alabama at Birmingham, Birmingham, Alabama‌‌, United States of America; Hirosaki University Graduate School of Medicine, JAPAN

## Abstract

The human genome contains approximately 3,200 near full-length autonomous human endogenous retroviral (HERV) loci capable of transcriptional activation through long terminal repeats. In our previous study, we developed the Window-based HERV Alignment (WHA) method to detect locus-specific HERV transcription from single-cell RNA sequencing (scRNA-seq) data. Using WHA, we previously identified patterns of autonomous HERV loci expression in monocytes from 12 patients with post-acute sequelae of COVID-19 (PASC). In the current study, we extended the analysis to peripheral blood mononuclear cells (PBMCs) from the same 12 PASC patients. In contrast to monocytes, no consistent HERV locus transcription patterns were found across B, T, natural killer, or dendritic cells. Subsequent analysis showed that the majority of expressed HERV loci were detected in CD14 + monocytes rather than CD16 + monocytes. Four HERV loci were identified in CD14 + monocytes and were absent in CD16 + , B, T, natural killer, and dendritic cells. One of the four, the HERV locus at Chr3:46,046,256–46,054,342 was also detected in bronchial lavage cells from COVID-infected individuals but not in normal lung or tuberculosis lung tissues. The HERV locus Chr3:46,046,256–46,054,342 is located within an intron of the host gene xCR1. xCR1 expression, a marker of mature dendritic cells, was detected in CD14 + PBMCs from 11 of 12 PASC patients. These findings suggest the presence of an atypical myeloid population in PASC and may inform future strategies to evaluate persistent immune dysfunction.

## 1. Introduction

Severe acute respiratory system (SARS) is now recognized as a multi-organ disease with a broad spectrum of short and long-term clinical manifestations [1–3]. Post-acute sequelae of COVID-19 (PASC), which affects approximately 10% of the patients, are characterized by persistent symptoms or delayed or long-term complications beyond 4 weeks from the onset of symptoms and can persist for up to two years in some individuals [1–3]. Although the mechanism(s) for the generation of PASC remain unknown, there is growing recognition of the ability of viral infections, including COVID-19, influenza, and Dengue virus, as well as bacterial infections (such as sepsis) to induce innate immune memory consisting of epigenetic modification of myeloid precursors that have the capacity for self-renewal and expansion following primary viral infection [4–10]. The development of effective detection methods for PASC will be needed to clarify whether these modified myeloid precursors and the subsequent mature immune cells play a role in the post clinical manifestation of SARS.

The human genome contains approximately 3,200 near full-length autonomous human endogenous retroviral (HERV) loci containing long terminal repeats capable of promoting RNA transcription [11–13]. In previous studies, we showed that the transcription of certain autonomous HERV loci in human monocytes varied in response to changes in the intracellular environment, resulting in individual-specific patterns of HERV expression [14,15]. To do this, we developed a method, Window-based HERV Alignment (WHA), that analyzes aligned HERV sequences using sequential, non-overlapping windows of defined nucleotide lengths and sequence depth [15]. We applied this method to scRNA-seq datasets from hospitalized COVID-19 patients, as well as from patients with influenza, dengue virus, or sepsis, and found distinct, individual-specific HERV expression patterns in monocytes [14]. We also used WHA to analyze 12 samples from patients with PASC collected 8 months after acute COVID-19. In contrast to the hospitalized acute COIVD-19 patients and those patients with influenza, dengue virus, or sepsis, WHA analysis of peripheral blood mononuclear cells (PBMCs) from the 12 PASC patients identified several shared patterns of HERV loci transcription in monocytes, with significantly greater numbers of extended HERV transcripts and increased sequence depth compared with monocytes from a panel of 30 healthy control PBMCs [14].

Circulating immune cells in the peripheral blood of PASC patients are known to exhibit chronic immune dysfunction [16–18]. Yin et al., who developed the 12 patient PASC scRNA-seq dataset used in our analyses, reported dysregulated T cells and an uncoordinated adaptive immune response in these patients [19]. In the current study, using WHA, we found that B, T, natural killer, and dendritic cell populations within PBMCs from these 12 PASC patients each exhibited distinct HERV loci compared with normal controls. However, these immune cell types did not show consistent HERV locus transcription patterns across patients, in contrast to monocytes. We identified HERV loci selectively detected in CD14 + monocytes but not in CD16 + cells or other PBMC populations. One HERV locus, detected in 11 of 12 PASC monocyte samples, was located within an intron of the host gene xCR1. Cells expressing both CD14, a marker of monocytes/macrophages, and xCR1, a marker of conventional dendritic cell (cDC), may represent an atypical circulating myeloid state that could contribute to persistent immune dysfunction in patients with PASC [20].

## 2. Methods

### 2.1. Datasets used in this study

In this study, we used publicly available scRNA-seq datasets from 1) 6 healthy individuals from Amrute et al. [21], 2) 2 healthy individuals from Derbois et al. [22], 3) 10 healthy individuals from Chen et al. [23], 4) 3 healthy individuals from Thompson et al. [24], 5) 4 healthy individuals from Lee et al. [25], 6) 5 healthy individuals from Yu et al. [26], 7) 3 healthy individuals from Liao et al. [27], and 8) lung tissue samples from 12 non-COVID-19 controls from Rooij et al [28].

For patients’ data, we obtained publicly available scRNA-seq datasets from 1) 12 PASC patients who consistently met the case definition for long COVID symptoms for at least 8 months following COVID-19 infection from Yin et al. [2,3,19], 2) lung tissue samples from 6 tuberculosis (TB) patients from Wang et al. [29], 3) 9 Bronchoalveolar lavage fluid (BAL) COVID-19 patients from Liao et al [27].

We used anonymized publicly available scRNA-seq datasets from NCBI repository. Any information on the individuals can be found in the primary publications. No new datasets were generated in our study.

### 2.2. scRNA-seq analysis

Single-cell RNA-seq datasets of peripheral blood mononuclear cells (PBMCs) were obtained from the NCBI SRA or GEO repositories, in either FASTQ or BAM format depending on data availability. When only BAM files were accessible, they were converted to FASTQ format using the 10x Genomics Cell Ranger software (v7.1.0) with the bamtofastq (v 1.4.1) (https://www.10xgenomics.com/support/software/cell-ranger/latest/analysis/inputs/cr-inputs-overview).

The resulting FASTQ files were processed using Cell Ranger count with default parameters and aligned to the GRCh38 human reference genome. Output files, including gene expression matrix, barcodes, and feature annotations, were imported into Seurat for downstream analysis.

Cell-type identification was performed using the Azimuth reference datasets for human PBMC and Human Lung v2 (https://satijalab.org/azimuth/) [30,31] to classify CD14^+^ and CD16^+^ monocytes and monocyte derived macrophages. The Azimuth-based workflow was used for reference-guided annotation rather than de novo cell-type discovery. Mapping quality was evaluated by summarizing the proportion of cells assigned to cell-type labels and the distribution of mapping confidence scores (mapping.score), which were used to assess annotation consistency across samples (**S1 Table**).

To extract sequence reads corresponding to each cell type, barcodes associated with annotated populations were used to filter BAM files with SAMtools (v 0.1.19). The resulting BAM files were converted to FASTQ format using bedtools (v 2.26.0).

### 2.3. Autonomous HERV expression analysis

The processed fastq files were subjected to Window-based HERV Alignment (WHA) analysis. For each sample, duplicate runs were generated and aligned against a reference set of 3,220 autonomous HERV loci using BWA (v 0.7.13) with a minimum percent match threshold of > 99%.

HERV loci with read depths below 3 or fewer than 9 usable windows were defined as negative, whereas loci with a read depth of at least 3 and 9 or more usable windows were considered positive HERV loci.

HERV profiles from patient and healthy control samples were compared to identify loci present in patient samples but absent in controls. During this filtering process, loci showing any detectable signal in control samples were excluded, ensuring that retained loci were not detected in healthy individuals but detected in at least one patient sample.

Differences in HERV loci detection across groups were visualized as heatmaps using R [32]. To compare HERV loci detected across monocyte subsets, we generated an UpSet plot in R to visualize shared and unique HERV loci between CD14+ and CD16 + monocyte populations.

### 2.4. Host Gene expression analysis

To further characterize host genes located within or near the HERV loci, we reprocessed the FASTQ files used in the WHA analysis. In this step, all reads were realigned to each corresponding gene reference sequence. For each group, we recorded the sequence depth and the number of good or usable windows for each gene. Statistical significance was assessed using one-way ANOVA followed by Tukey’s HSD test (P < 0.05) for each gene.

## 3. Results

In a previous study, we described the Window-based HERV Alignment (WHA) method, in which sequence reads from scRNA-seq data are aligned to 3,200 autonomous HERV loci using non-overlapping windows of defined nucleotide lengths [14,15] (**Supplementary Methods,** S1 Fig). Usable windows were defined as those with a read depth of 3 or greater and at least 9 good/usable windows per locus, corresponding to extended (i.e., longer) HERV transcript expression. In contrast, HERV loci designated as negative (8 or fewer windows) did not meet the required sequence read depth or number of good/usable windows as determined by WHA.

In quiescent, normal cells, only a subset of autonomous HERV loci is transcriptionally active, whereas most remain silenced [33–35]. To establish a reference baseline, we generated a control dataset consisting of scRNA-seq data from 30 healthy individuals across multiple studies. HERV loci showing any detectable signal in control samples were excluded from further analysis [14,15].

**Fig 1:**
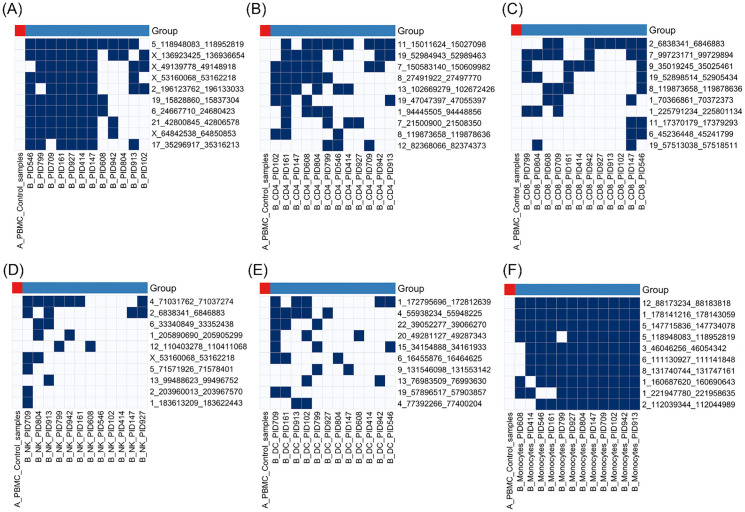
Top 10 shared HERV loci across cell types in PASC patients‌‌ versus healthy controls.

We then analyzed PBMCs from 12 PASC patients using WHA and compared them with PBMCs from 30 healthy controls to identify HERV loci detected in patient samples but not in controls. To prioritize the most consistent loci, each HERV locus was scored based on the number of patient samples in which it was detected, and the top 10 loci with the highest frequencies were selected for visualization (**Fig 1**; S2 Table).

The top 10 HERV loci with the highest frequency of detection across 12 PASC patients and 30 healthy controls are shown. Panels (A–F) show results for B cells, CD4^+^ T cells, CD8^+^ T cells, NK cells, dendritic cells, and monocytes, respectively. Blue squares represent detected HERV loci (≥ 9 usable windows and read depth ≥ 3), while white squares indicate HERV loci not detected. Ranking was based on the number of PASC samples in which each locus was detected, with the most frequently detected loci displayed at the top. Healthy controls are shown as a single merged column for visualization clarity; however, control samples were analyzed at the individual-donor level, and loci showing any detectable signal in any healthy donor were excluded from prioritization. This figure provides a descriptive summary of locus-level detection patterns and is not intended for inferential statistical comparisons. The underlying matrices used to derive the top 10 loci for each cell type are provided in S2 Table (A-F).

Common HERV loci patterns within the majority of the 12 PASC patients were detected within monocytes of the 12 PASC patients [14]. We next analyzed HERV loci expression in B, T, natural killer (NK), and dendritic cells using the same scRNA-seq datasets from 12 PASC patients and 30 normal controls. In contrast to monocytes, no HERV loci were detected across all 12 PASC patients in these other immune cell types. One HERV locus was detected in B cells in 11 of 12 PASC patients, whereas 6 loci were detected in monocytes in 11 of 12 patients.

**Fig 2:**
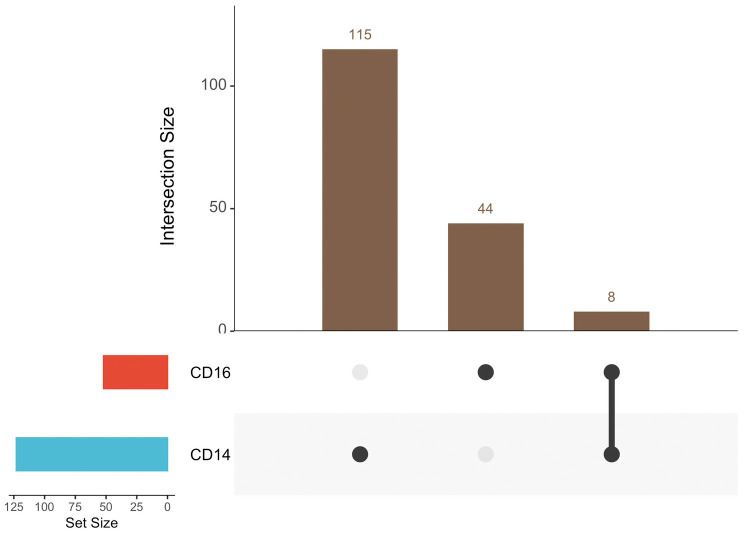
Detection of the HERV locus Chr3:46,046,256–46,054,342 in monocyte subsets.

To further delineate the monocyte population, we next analyzed CD14^+^ and CD16^+^ subsets to determine which population contributes to the observed HERV loci (**Fig 2**, S3 Table).

The distribution of positive HERV loci across CD14⁺ and CD16 ⁺ monocyte populations from PASC samples (*n* = 12) is shown in an UpSet plot. The PASC-associated locus Chr3:46,046,256–46,054,342 (red line) was detected in CD14 ⁺ monocytes, with no positive HERV loci detected in CD16 ⁺ cells. S3 Table provides the values used to generate this UpSet plot.

Overall, most HERV loci were detected in CD14 + monocytes within PBMCs from PASC patients (**Fig 2**, S3 Table**).** Four HERV loci were detected only in CD14 + monocytes and not in B, T, natural killer, or dendritic cells: Chr1:178,141,216−178,143,059, Chr3:46,046,256−46,054,342, Chr6:111,130,927−111,141,848, and Chr12:88,173,234−88,183,818 (S4 Table)**.**

**Fig 3:**
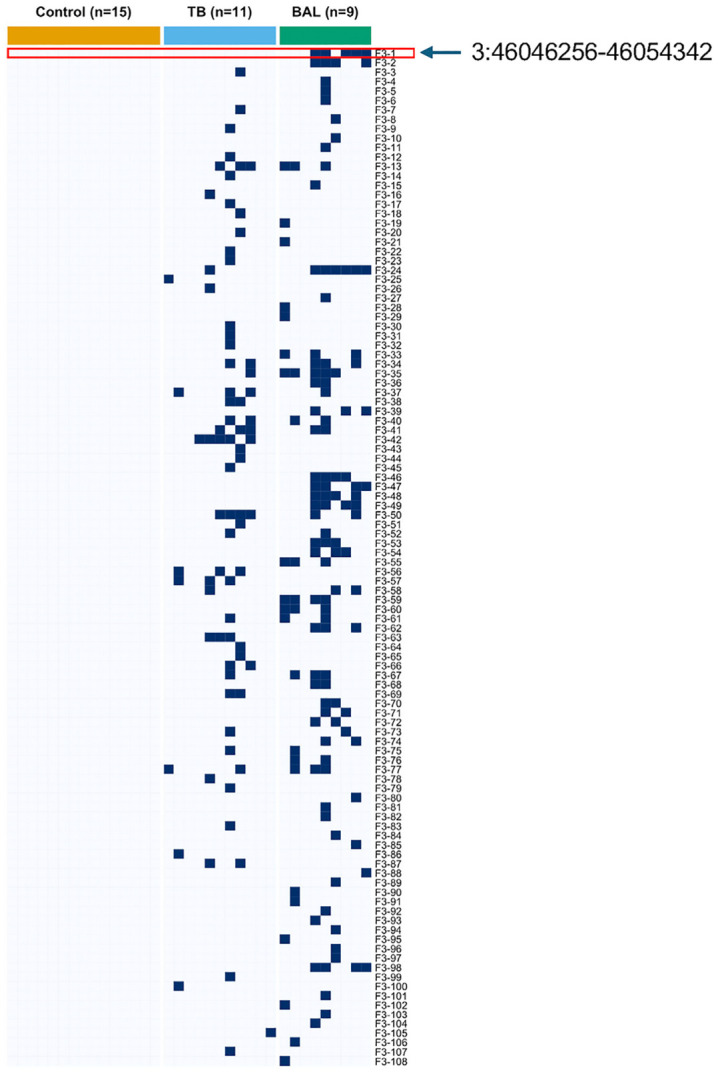
Distribution of positive HERV loci within TB, BAL, and control datasets.

Proinflammatory and monocyte-derived macrophages are increased in the lungs during severe COVID-19 infections [36]. We next assessed the HERV locus expression in lung-associated datasets including healthy controls, BAL from COVID-19 patients, and TB lung tissue (**Fig 3**, S5 Table).

The presence (“+”) or absence (“–”) of HERV loci is shown across samples from 11 tuberculosis (TB) patients, 9 bronchoalveolar lavage (BAL) samples from COVID-19 patients, and 15 healthy controls. The HERV locus Chr3:46,046,256–46,054,342 was detected in BAL samples from COVID-19 patients but was not detected in TB and healthy control datasets. Blue squares indicate detected HERV loci (≥ 9 usable windows and read depth ≥ 3). The presence/absence data used to generate this heatmap are provided in S5 Table.

We found that the HERV loci, Chr1:178,141,216−178,143,059, Chr12:88,173,234−88,183,818, and Chr6:111,130,927−111,141,848 were detected in normal lung tissue. In contrast, the HERV locus Chr3:46,046,256−46,054,342 was not detected in normal lung tissue or in lung tissue from 12 patients with tuberculosis (TB) (**Fig 3**, S5 Table). Importantly, the HERV locus Chr3:46,046,256−46,054,342 was detected in 5 of 10 BAL samples, specifically within monocyte derived macrophage populations. Within the BAL dataset, samples C143, C145, C148, C149, and C152 (all classified as severe COVID-19) were positive for this locus, whereas none of the mild BAL cases (C141, C142, C144) were HERV positive (**S5 Table**). The overall patterns of HERV loci in the BAL datasets were more heterogeneous, with a greater diversity of detected loci across samples.

**Fig 4:**
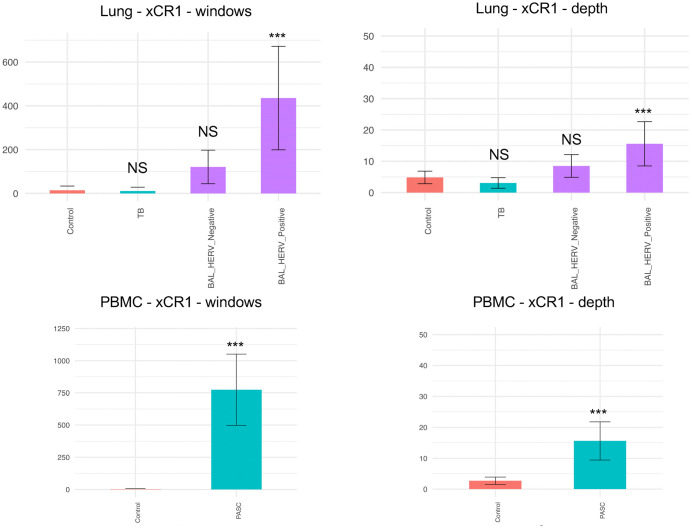
Comparison of xCR1 window counts and sequence depth between sample groups.

To further substantiate these findings, we analyzed the expression of the host gene xCR1associated with the HERV locus Chr3:46,046,256−46,054,342 in PBMC samples from PASC patients and lung tissue, BAL, and TB datasets (**Fig 4**, S6 Table).

Bar plots showing the number of usable windows and sequence depth for xCR1 across tissue-matched groups. PBMC datasets include healthy controls and PASC patients, while lung datasets include tuberculosis (TB) samples and bronchoalveolar lavage (BAL) samples stratified into HERV-negative and HERV-positive subsets. Within each tissue type, both window counts and read depths were significantly higher in PASC compared to healthy PBMC controls and in BAL HERV-positive samples compared to lung controls (ANOVA with Tukey’s HSD). No significant differences were observed in TB or BAL HERV-negative groups within lung datasets. These results suggest an association between HERV activity and xCR1 expression in COVID-19–related samples. Significance levels are indicated as ***P < 0.001. The numerical window and depth values used for this analysis are provided in S6 Table.

Both the average window numbers and sequence depths for xCR1 were significantly higher in PASC and BAL HERV-positive samples compared with controls (*P < 0.001*, ANOVA with Tukey’s HSD). In contrast, TB and BAL HERV-negative samples showed no significant differences from controls (NS), indicating that elevated xCR1 expression was associated with COVID-19–related HERV positivity. However, analysis of the HERV locus Chr3:46,046,256−46,054,342 across B, T, NK, and dendritic cell populations from PASC PBMCs showed no detectable signal in these cell types (S2 Table**).** Together, these results indicate that expression at the HERV locus Chr3:46,046,256−46,054,342 is associated with increased xCR1 expression in CD14 + cells from both BAL samples of COVID-19-infected patients and circulating PBMCs from PASC patients.

## 4. Discussion

In this study, we used WHA analysis of scRNA-seq data from 12 individuals with PASC to determine the expression patterns of autonomous HERV loci. Our results show that the pattern of HERV loci transcription in the 12 PASC patients was shared within the majority of peripheral monocytes, but not in B, T, NK, or dendritic cells. The circulating monocytes exhibited unique features of co-expression of CD14 and xCR1, which is atypical for healthy circulating myeloid cells and may reflect an altered immune state in patients with PASC.

These findings extend our previous work, in which WHA analysis of PBMC scRNA-seq datasets from 12 PASC patients identified HERV loci expression in monocytes [14]. In the current study, we further show that these patterns are unique to monocytes and are not found in B, T, NK, or dendritic cells from the same PASC patients. Further analysis of the circulating myeloid cells in the PASC patients revealed the unexpected finding that transcriptional activity at the HERV locus Chr3:46,046,256–46,054,342 was detected in CD14 + monocytes in 11 of 12 PASC patients. This HERV locus is located within the first intron of the host gene xCR1, a defining marker of the conventional dendritic cell 1 (cDC1) subset [20]. We did not detect activity at the HERV locus Chr3:46,046,256−46,054,342 or elevated xCR1 expression in dendritic cells from PASC patients, although we did observe other HERV loci in dendritic cells compared with controls, consistent with previous reports describing persistent dendritic-cell dysregulation for up to 7 months following COVID-19 infection [37].

The positive HERV locus Chr3:46,046,256–46,054,342 was detected in BAL samples, which are known to contain monocyte-derived macrophages from patients with severe COVID-19. Importantly, the expression of the positive HERV locus Chr3:46,046,256−46,054,342 was found mainly in BAL from severe COVID-19-infected patients. Consistent with this result, a previous genome-wide association study (GWAS) identified the 3p21.31 gene cluster, which contains the HERV-xCR1 locus, as a genetic susceptibility region for severe COVID-19 [38]. Thus, cells expressing both CD14, a marker of monocytes/macrophages, and xCR1, a marker of conventional dendritic cells, represent an atypical circulating myeloid state in PASC patients. We acknowledge that a limitation of our analysis is that we examined only a single PASC dataset. As previously discussed, the selection criteria for these 12 PASC patients may have been biased toward individuals with unresolved symptoms [2,3,14,19]. Thus, the observation that all 12 patients exhibited similar HERV locus detection patterns may, in part, reflect this selection bias. Further studies are needed to determine whether HERV locus Chr3:46,046,256−46,054,342 is detectable in independent PASC cohorts with similar clinical characteristics.

## 5. Conclusion

A key observation from our analysis of the 12 PASC patients was that HERV loci were consistently detected in circulating monocytes from PASC patients compared with those observed in other immune cell types. It is known that circulating monocytes have a limited half-life, likely due to their migration into tissues [39]. Thus, it is unlikely that monocytes from the acute COVID infection phase would persist in the circulation of PASC patients 8 months after the primary infection. Previous studies have demonstrated that COVID-19 infection can generate myeloid precursor populations with self-renewal capacity, providing a potential source of long-lived progenitors capable of sustaining these transcriptional patterns [10,40]. The presence of CD14 + cells with xCR1 + expression in the circulation of the 12 PASC patients is consistent with the possibility that these cells arose from expanded myeloid progenitors that had been altered as a result of COVID-19 infection [10,40]. An additional feature of these progenitor populations may include epigenetic remodeling within the myeloid precursor compartment [10]. In our previous study, we identified in all 12 PASC patients a HERV located in an intron of the Janus kinase and microtubule interacting protein 2 (JAKMIP2) gene. Similar to xCR1, both the HERV and JAKMIP2 transcript showed increased expression relative to healthy controls [14]. Together, these findings support that these atypical myeloid cells with expression at the HERV locus Chr3:46,046,256–46,054,342 and xCR1 may arise from epigenetically modified myeloid precursors generated during COVID-19 infection [40–43].

## Supporting information

S1 Fig Overview of scRNA-seq processing and WHA-based HERV transcription analysis workflow. Publicly available scRNA-seq datasets were downloaded from GEO or SRA, converted to FASTQ when required, and aligned to the GRCh38 reference genome using Cell Ranger. Cell-type annotation was performed using Azimuth reference datasets (PBMC and Lung), followed by barcode-based extraction of reads from defined immune cell subsets. Filtered FASTQ files were analyzed using Window-based HERV Alignment (WHA) with 3,220 autonomous HERV loci as references. HERV loci were defined as transcriptionally positive when sequence depth ≥3 and ≥9 usable windows were detected and were used for downstream analyses.
(TIF)

S1 Table Azimuth annotation performance metrics for all analyzed samples, including cell counts, assignment rates, and mapping confidence scores. (XLSX)

S2 Table (A-F) Summary of positive HERV loci across all samples for six cell types (B, CD4 ⁺ , CD8 ⁺ , NK, DC, and CD14 ⁺ monocytes). Each column represents an individual sample, and each row corresponds to a HERV locus; the rightmost “+” counts were used to identify the top 10 loci shown in Fig 1.
(XLSX)

S3 Table Presence (“+”) or absence (“–”) of HERV loci across CD14⁺ and CD16 ⁺ monocyte subsets in PASC samples. Rows represent HERV loci, and columns represent individual samples. Detection was defined as ≥9 usable windows and read depth ≥3.
(XLSX)

S4 Table Number of PASC patients with detected HERV loci across PBMC cell types, including monocytes (CD14 ⁺ , CD16⁺) and lymphoid populations (B, CD4 ⁺ T, CD8 ⁺ T, NK, and dendritic cells). Values indicate the number of patients (out of 12 PASC individuals) with detectable signals for each locus in each cell type.
(XLSX)

S5 Table Presence (“+”) or absence (“–”) of HERV loci across macrophage samples from non-COVID controls, tuberculosis (TB), and COVID-19 bronchoalveolar lavage (BAL) datasets. Rows represent HERV loci, and columns represent individual samples. Detection was defined as ≥9 usable windows and read depth ≥3.
(XLSX)

S6 Table Window counts and sequence depth for XCR1 across PBMC and lung macrophage samples. Values are shown for individual samples, with averages provided for each group. The presence (“+”) or absence (“–”) of the HERV locus Chr3:46,046,256–46,054,342 is indicated. Detection was defined as ≥9 usable windows and read depth ≥3.
(XLSX)

S7 File Supplementary methods. Detailed supplementary methods used in this study.
(PDF)
