# Supplementary figures and images for: Shared autonomous HERV loci transcription identifies a unique circulating CD14+-xCR1+ mononuclear cell phenotype in a patient group with post-acute sequelae of COVID-19
Source: PLoS One. 2026 May 19;21(5):e0349350. doi: 10.1371/journal.pone.0349350 (PMC13186335; doi:10.1371/journal.pone.0349350)

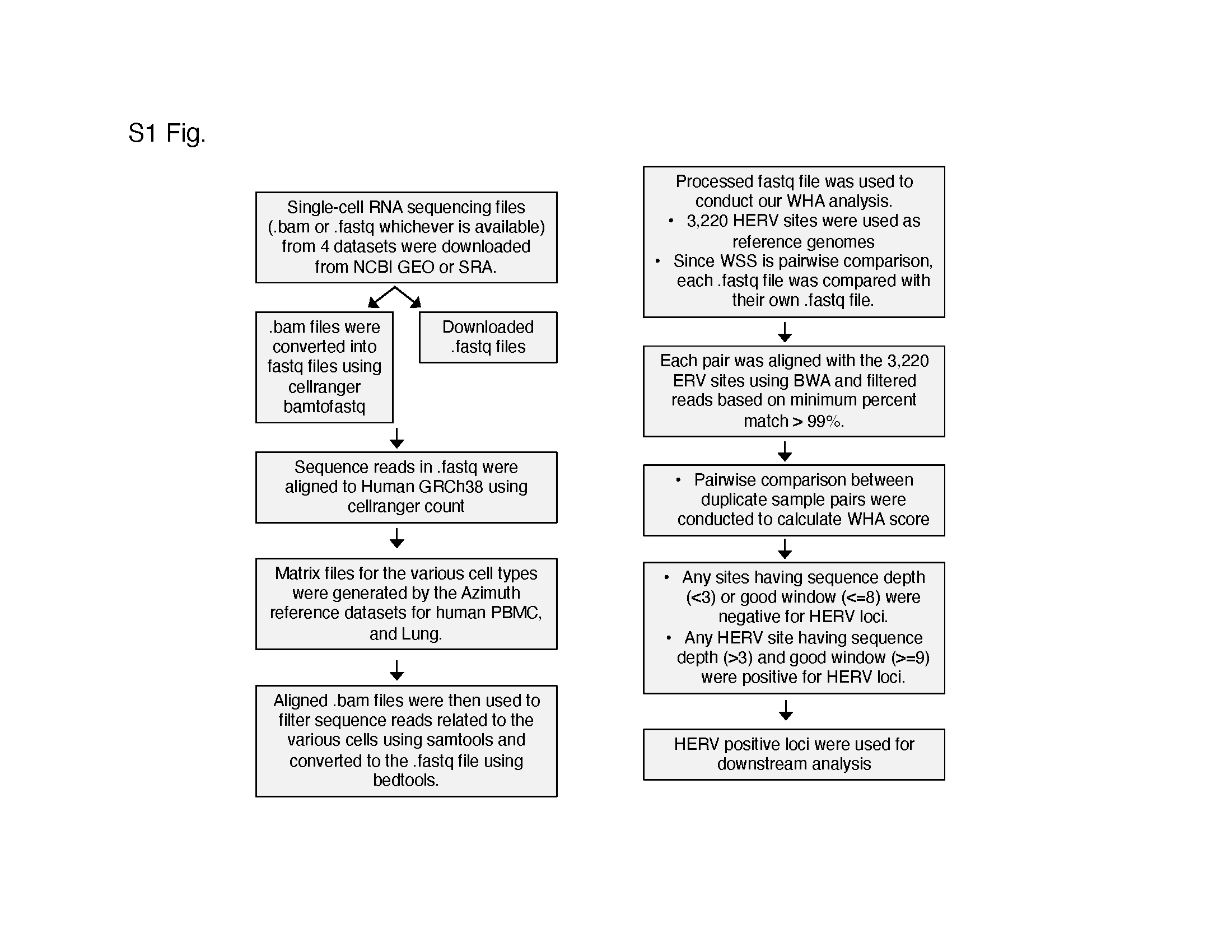

Supplement: S1 Fig — Publicly available scRNA-seq datasets were downloaded from GEO or SRA, converted to FASTQ when required, and aligned to the GRCh38 reference genome using Cell Ranger. Cell-type annotation was performed using Azimuth reference datasets (PBMC and Lung), followed by barcode-based extraction of reads from defined immune cell subsets. Filtered FASTQ files were analyzed using Window-based HERV Alignment (WHA) with 3,220 autonomous HERV loci as references. HERV loci were defined as transcriptionally positive when sequence depth ≥3 and ≥9 usable windows were detected and were used for downstream analyses. (TIF) [file pone.0349350.s001.tif]
