# Supplementary material for: Shared autonomous HERV loci transcription identifies a unique circulating CD14+-xCR1+ mononuclear cell phenotype in a patient group with post-acute sequelae of COVID-19
Source: PLoS One. 2026 May 19;21(5):e0349350. doi: 10.1371/journal.pone.0349350 (PMC13186335; doi:10.1371/journal.pone.0349350)
Supplement: S7 File — Detailed supplementary methods used in this study. (PDF) [file pone.0349350.s008.pdf]

Supplementary Methods

Shared autonomous HERV loci transcription identifies a unique circulating CD14+-xCR1+ mononuclear cell phenotype in a patient group with post-acute sequelae of COVID-19

Hyunmin Koo and Casey D. Morrow*

Window-based SNV similarity (WSS) analysis.

In this study, we used a WHA tool, which was modified from our previous tool, called WSS. For the WSS analysis, sequence reads from each sample were aligned to the 93 microbial reference genomes which were previously established based on the HMP dataset [1, 2] using the Burrows-Wheeler aligner (BWA) tool [3]. Each sample was analyzed for multi-sample SNVs relative to the provided reference genome using the Genome Analysis Toolkit (GATK) [4]. The resulting multi-sample Variant Call Format (VCF) files were utilized for pairwise comparisons between all possible pairs of samples. This was done to determine the overall genome-wide SNV similarity for each microbial species. Samples with sequence coverage below 30% and sequence depth less than 3.5 against their given reference genome were excluded from the pairwise comparisons [1, 5-11]. In order to identify related strains, the WSS score was compared to a previously established cut-off value from our earlier study (For related strain pairs: WSS score>cut-off; for unrelated strain pairs: WSS score<cut-off) [1, 12].

In WSS, a window is defined as similar if the SNV pattern is exactly the same between compared two samples or no SNV is present in both samples. Good (usable) windows are defined when each window had more than 50% of the bases having a satisfied coverage and depth value compared to cut-off values.

Window-based HERV Alignment (WHA) analysis.

In this study, we used the Window-based HERV Alignment (WHA) analysis. [13, 14]. For WHA, the original microbial reference genomes were replaced with a custom reference composed of 3,200 autonomous HERV loci [13-17]. We also implemented additional code modules to incorporate 10X Genomics hashtag-derived cell-type assignments, allowing integration with the Azimuth reference database for cell-type prediction and subsequent filtering of cell-type-specific sequence reads prior to WHA preprocessing. The same alignment tool was used to map HERV

reference loci with a minimum percent match threshold of 99%. Window size parameters were adjusted from 100 base pairs to 50 base pairs, and the sequence depth cutoff was lowered from 5 to 3. In addition, a new positivity criterion was applied, in which loci with more than 8 good/usable windows (corresponding to >= 400 bp of aligned sequence) were designated as positive HERV loci. Further details of the WHA algorithm and filtering workflow can be found in our previous publications [13, 14]

References

1. Kumar, R., et al., *Identification of donor microbe species that colonize and persist long term in the recipient after fecal transplant for recurrent Clostridium difficile.* NPJ biofilms and microbiomes, 2017. **3**(1): p. 12.
2. Schloissnig, S., et al., *Genomic variation landscape of the human gut microbiome.* Nature, 2013. **493**(7430): p. 45-50.
3. Li, H. and R. Durbin, *Fast and accurate long-read alignment with Burrows–Wheeler transform.* Bioinformatics, 2010. **26**(5): p. 589-595.
4. Van der Auwera, G.A., et al., *From FastQ data to high confidence variant calls: the Genome Analysis Toolkit best practices pipeline.* Curr Protoc Bioinformatics, 2013. **43**: p. 11.10.1-33.
5. Koo, H., D.K. Crossman, and C.D. Morrow, *Strain Tracking to Identify Individualized Patterns of Microbial Strain Stability in the Developing Infant Gut Ecosystem.* Frontiers in Pediatrics, 2020. **8**.
6. Koo, H., et al., *Individualized recovery of gut microbial strains post antibiotics.* NPJ Biofilms Microbiomes, 2019. **5**: p. 30.
7. Koo, H., et al., *Sharing of gut microbial strains between selected individual sets of twins cohabitating for decades.* PLOS One, 2019. **14**(12): p. e0226111.

8. Koo, H., et al., *An individualized mosaic of maternal microbial strains is transmitted to the infant gut microbial community.* Royal Society Open Science, 2020. **7**: p. 192200.
9. Koo, H. and C.D. Morrow, *Perturbation of the human gastrointestinal tract microbial ecosystem by oral drugs to treat chronic disease results in a spectrum of individual specific patterns of extinction and persistence of dominant microbial strains.* PLOS One, 2020. **15**(12): p. e0242021.
10. Koo, H. and C.D. Morrow, *Bacteroidales-specific antimicrobial gene analysis identifies gastrointestinal tract reservoirs of microbial sub strains selected for fecal dominance.* PREPRINT (Version 1) available at Research Square, 2022.
11. Koo, H. and C.D. Morrow, *Time series strain tracking analysis post fecal transplantation identifies individual specific patterns of fecal dominant donor, recipient, and unrelated microbial strains.* Plos one, 2022. **17**(9): p. e0274633.
12. Kumar, R., et al., *New microbe genomic variants in patients fecal community following surgical disruption of the upper human gastrointestinal tract.* Human Microbiome Journal, 2018. **10**: p. 37-42.
13. Koo, H. and C.D. Morrow, *Shared and unique patterns of autonomous human endogenous retrovirus loci transcriptomes in CD14+ monocytes from individuals with physical trauma or infection with COVID-19.* Retrovirology, 2024. **21**(1): p. 17.
14. Koo, H. and C.D. Morrow, *Amplification of select autonomous HERV loci and surrounding host gene transcription in monocytes from patients with post-acute sequelae of COVID-19.* Frontiers in Immunology, 2025. **16**: p. 1621657.
15. Lander, E.S., et al., *Erratum: Initial sequencing and analysis of the human genome: international human genome sequencing consortium (Nature (2001) 409 (860-921)).* Nature, 2001. **412**(6846): p. 565-566.
16. Vargiu, L., et al., *Classification and characterization of human endogenous retroviruses; mosaic forms are common.* Retrovirology, 2016. **13**: p. 1-29.

17. Tokuyama, M., et al., *ERVmap analysis reveals genome-wide transcription of human endogenous retroviruses.* Proceedings of the National Academy of Sciences, 2018. **115**(50): p. 12565-12572.
